# Hyperopia: a meta-analysis of prevalence and a review of associated factors among school-aged children

**DOI:** 10.1186/1471-2415-14-163

**Published:** 2014-12-23

**Authors:** Victor Delpizzo Castagno, Anaclaudia Gastal Fassa, Maria Laura Vidal Carret, Manuel Augusto Pereira Vilela, Rodrigo Dalke Meucci

**Affiliations:** Department of Specialized Medicine – Ophthalmology, Federal University of Pelotas, Rua Marechal Deodoro, 1160, Centro, 96020-220 Pelotas, RS Brazil; Department of Social Medicine, Rua Marechal Deodoro, 1160, Centro, 96020-220 Pelotas, RS Brazil; Department of Social Medicine, Federal University of Pelotas, Avenida Duque de Caxias, 250, Fragata, 96001-970 Pelotas, RS Brazil

**Keywords:** Children, Cross-Sectional Studies, Hyperopia, Longitudinal Studies, Prevalence

## Abstract

**Background:**

Studies show great variability in the prevalence of hyperopia among children. This study aimed to synthesize the existing knowledge about hyperopia prevalence and its associated factors in school children and to explore the reasons for this variability.

**Methods:**

This systematic review followed PRISMA guidelines. Searching several international databases, the review included population- or school-based studies assessing hyperopia through cycloplegic autorefraction or cycloplegic retinoscopy. Meta-analysis of hyperopia prevalence was performed following MOOSE guidelines and using the random effects model.

**Results:**

The review included 40 cross-sectional studies. The prevalence of hyperopia ranged from 8.4% at age six, 2-3% from 9 to 14 years and approximately 1% at 15 years. With regard to associated factors, age has an inverse association with hyperopia. The frequency of hyperopia is higher among White children and those who live in rural areas. There is no consensus about the association between hyperopia and gender, family income and parental schooling.

**Conclusion:**

Future studies should use standardized methods to classify hyperopia and sufficient sample size when evaluating age-specific prevalence. Furthermore, it is necessary to deepen the understanding about the interactions among hyperopic refractive error and accommodative and binocular functions as a way of identifying groups of hyperopic children at risk of developing visual, academic and even cognitive function sequelae.

## Background

Hyperopia in childhood, particularly when severe and/or associated with accommodative and binocular dysfunctions, may be a precursor of visual motor and sensory sequelae such as accommodative esotropia, anisometropia and unilateral or bilateral amblyopia [1, 2]. Children with hyperopia may also present symptoms related to asthenopia while reading.

Studies have also shown that axial length (AL) of the eye or the relation between AL and corneal curvature (CC) radius plays an important role in the variability of hyperopic spherical equivalent refraction (SE) [3–8]. Utermen observed that after logistic regression, the combination of AL and CC contributed to explaining 60.9% of variability in hyperopic SE among children aged 3 to 14 years on average [5].

Although there are several studies on hyperopia, so far there has been no systematic review of the subject. This systematic review aims to synthesize existing knowledge about the hyperopia prevalence and associated factors among children, followed by a meta-analysis of hyperopia prevalence. This synthesis may help in the design of appropriate public policies to correct hyperopia in children.

## Methods

### Systematic review

The literature search was performed on MEDLINE (PubMed), Scielo, Bireme, Embase, Cochrane Library, Clinical Trials registration website and WHO databases. The following descriptors were used: refractive errors, hyperopia, prevalence and children, limited to keywords or words in the title or abstract, in either their isolated or combined form. The searches were limited to the 0-18 age range.

**Figure 1:**
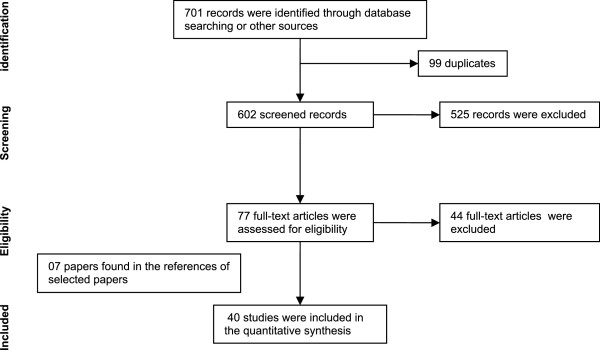
**Flow of information through the different phases of the systematic review.**

A total of 701 records were identified and screened (including theses, journals, articles, books, book chapters and institutional reports) relating to hyperopia prevalence in children up to 18 years old. 99 of these articles were duplicated. Population-based or school-based studies assessing hyperopia through cycloplegic autorefraction or cycloplegic retinoscopy were included. 525 papers were excluded owing to their focus on: specific populations as well as publications about refractive errors in subjects with eye diseases (amblyopia, strabismus, glaucoma, corneal abnormalities, chromatic aberrations, accommodative and binocular dysfunction and asthenopia); other specific clinical diseases or conditions (intellectual disability, cerebral palsy, dyslexia and prematurity); ophthalmology/optometry outpatients; genetic and/or congenital alterations; before and/or after examinations, clinical and/or surgical treatment; cost-benefit research and geographically isolated populations. A further 44 articles were excluded due to: non-random sample of the general population and schools; determination of refractive error without cycloplegia; cycloplegia only in children with low vision; hyperopia based only on visual acuity testing, studies without specific cut-off for hyperopia, samples excluding children that were already in eye care treatment, samples based on records of clinics or mobile clinics, very small and stratified samples. 07 papers found in the references of the selected articles were included (Figure 1).

### Meta-analysis

Meta-analysis was undertaken regarding prevalence of moderate hyperopia at specific ages in 6 to 15 year-olds. Out of a total of 21 articles on hyperopia prevalence at specific ages (Table 1), three had losses of more than 20% and six did not report their response rates. Fotouhi’s study showed prevalence estimates significantly different to all the other studies in various age groups, and its inclusion in the meta-analysis resulted in a statistically significant heterogeneity test (p < 0.05). Based on the heterogeneity assumption for the effect summary, Fotouhi’s study was characterized as an outlier and excluded from the meta-analysis. Following this, the heterogeneity test produced a p-value > 0.1 in all specific ages [9]. Thus the meta-analysis was based on 11 studies assessing moderate hyperopia taking ≥ +2.00D as the cut-off point and a response rate greater than 80% (Table 1).

**Table 1:** **Hyperopia prevalence among children in the analyzed studies**

Author (Year)	***N***	Age range	Hyperopia definition	Response Rate (%)	Prevalence (%)	95% CI	Age specific prevalence (95% CI)
Location							
			SE				
Zhao (2000) [10]	5884	5-15 years	≥ + 2.00 D	95.9	2.7	Not available	Males:
			RESC				5 years: 8.8% (2.4 – 15.2)
Shunyi District, China			ca				15 years: less than 2%
							Females:
							5years: 19.6% (8.1 – 31.0)
							15 years: less than 2%
He (2004) [11]	4364	5-15 years	≥ + 2.00 D	86.4	4.6	4.4 – 4.9	5 years: 17.0% (12.8 – 21.3)
			RESC				6 years: 10.7% ( 6.4 – 15.1)
Guangzhou, China			ca				7 years: 4.0% (1.3 – 6.7)
							8 years: 7.1% (3.9 – 10.4)
							9 years: 3.8 % (2.0 – 5.6)
							10 years: 4.6% (2.1 – 7.1)
							11 years: 3.5% (1.7 – 5.6)
							12 years: 2.0% (0.5 – 3.6)
							13 years: 3.4 % (1.6 – 5.2)
							14 years: 1.2% (0.3 – 2.1)
							15 years: 0.5% (0.0 – 1.3)
Fan (2004) [12]	7560	5-16 years	≥ + 2.00 D	Not stated	4.0	Not available	Not available
			Right eye				
			ca				
Hong Koong, China							
Zhan (2000) [13]	Xiamen city: 132	6-7 years	≥ + 2.00 D	Not stated	Xiamen city: 3.0	0.8 – 7.8	Not available
	Xiamen		Right eye		Xiamen countryside: 1.9	1.4 – 2.3	
			ca		Singapore: 2.7	0.8 – 6.9	
Xiamen city, Xiamen Countryside and Singapore, China	countryside: 104						
	Singapore: 146						
Pi (2010) [14]	3070	6-15 years	≥ + 2.00 D	88.50	3.26	2.6 – 3.9	6 years: 9.21% (5.5 – 12.9)
Yong Chuan District, Western China			At last one eye was hyperopic				7 years: 7.7% (4.7 – 10.6)
							8 years: 5.3% (2.9 – 7.7)
			cr				9 years 3.1% (1.3 – 4.9)
							10 years: 3.5% (1.6 – 5.5)
							11 years: 1.2% (0.0 – 2.5)
							12 years: 0.7% (0.0 – 1.6)
							13 years: 0.3% (0.0 – 1.0)
							14 years: 1.1% (0.0 – 2.2)
							15 years: 0.9% (0.0 – 2.1)
He (2007) [15]	2454	12-18 years	≥ + 2.00 D	97.6	1.20	0.8 – 1.6	13 years: 0.9% (0.1 – 3.1)
			RESC				14 years: 1.5 % (0.5 – 2.5)
			ca				15 years: 1.3 % (0.5 – 2.2)
Yangxi County, China							16 years: 1.0% (0.3 – 2.5)
							17 years: 0.0
Saw (2006) [16]	Malaysia:	7-9 years	≥ +2.00D	83.3	Malaysia:2.9	1.9 – 3.8	Malaysia (N = 1752)
							7 years: 5.0% (3.0 – 7.0)
							8 years: 2.0% (0.7 – 3.3)
Kuala Lumpur, Malaysia	1752		RESC		Singapore: 1.7	1.2 – 2.4	9 years: 1.6% (0.4 – 2.8)
Singapore	Singapore:1962		ca				Singapore (N = 1962)
							7 years: 2.1% (1.3 – 3.3)
							8 years: 1.9% (1.0 – 3.3)
							9 years: 0.8% (0.2 – 2.1)
Goh (2005) [17]	4634	7-15 years	≥ + 2.00 D	83.8	1.6	1.1 – 2.1	7 years: 5.0% (3.0 – 7.0)
			RESC				8 years: 2.0% (0.7 – 3.3)
			ca				9 years: 1.6% (0.4 – 2.8)
							10 years: 1.4 % (0.1 – 2.6)
							11 years: 0.9 % (0.0 – 2.6)
Gombak District, Malaysia							12 years: 0.6% (0.0 – 1.2)
							13 years: 0.5% (0.0 – 1.1)
							14 years: 0.0
							15 years: 0.9% (0.0 – 1.9)
Pokharel (2000) [18]	5067	5-15 years	≥ + 2.00 D	Not stated	2.1	Not available	Not available
			RESC				
			ca				
Mechi Zone, Nepal							
Gao (2012) [19]	5527	12-14 years	≥ + 2.00 D	89.8	Urban: 1.4	0.1– 1.7	Urban:
			At last one eye was hyperopic		Rural: 0.4	0.2 – 0.6	12 years: 0.7% (0.4 – 1.0)
							13 years: 0.7% (0.4 – 0.9)
Phnom Penhn and Kandal Provinces, Cambodia			cr				14 years: 0.8% (0.3 – 1.3)
Casson (2012) [20]	2899	6-11 years	≥ + 2.00 D	87.0	2.8	1.9 – 3.7	6 years: 3.1% (1.7 – 5.1)
			RESC				11years: 1.1% (0.3 – 2.9)
			cr				
Vientiane Province, Lao PDR							
Murthy (2002) [21]	6447	5-15 years	≥ + 2.00 D	92	7.4	6.0 – 8.8	5 years: 15.6 % (11.0 – 20.2)
			RESC				
			cr				
New Delhi, India							6 years: 13.0% (9.1 – 16.8)
							7 years: 10.7% (7.0 – 14.2)
							8 years: 8.5% (5.9 – 11.2)
							9 years: 6.6% (3.7 – 9.5)
							10 years: 5.2% (2.4 – 8.1)
							11 years: 7.8% (4.7 – 10.8)
							12 years: 5.0% (3.5 – 6.5)
							13 years: 3.3% (1.7 – 4.9)
							14 years: 4.4% (2.4 – 6.5)
							15 years: 3.9% (2.1 – 5.7)
Dandona (2002) [22] Mahabubnagar, Andhra Pradesh, India	4074	7-15 years	≥ + 2.00 D	92.3	0.68	0.4 – 1.0	Rural:
			At last one eye was hyperopic				7 years: 0.7% (0.0 – 1.2)
							8 years: 0.3% (0.0 – 0.8)
							9 years: 0.4% (0.0 – 1.0)
			cr				10 years: 1.2% (0.1 – 2.3)
							11 years: 1.6% (0.4 – 2.8)
							12 years: 0.8% (0.0 – 1.5)
							13 years: 0.6% (0.0 – 1.4)
							14 years: 0.3% (0.0 – 1.1)
							15 years: 1.1% (0.0 – 2.6)
Uzma (2009) [23]	Urban: 1789	7-15 years	≥ + 2.00 D	Not stated	Urban: 3.3	1.8 – 4.8	Urban:
Hyderabad, India	Rural: 1525		At last one eye was hyperopic		Rural: 3.1	1.7 – 4.5	7 years: 4.6% (2.6 – 6.6)
			ca				8 years: 2.0% (0.4 – 3.6)
							9 years: 1.7% (0.8 – 2.6)
							10 years: 1.3% (0.5 – 2.1)
							11 years: 2.2% (0.9 – 3.1)
							12 years: 0.4% (0.0 – 0.8)
							13 years: 0.2% (0.0 – 0.4)
							14 years: 0.0
							15 years: 0.4% (0.0 – 0.8)
							Rural:
							7 years: 9.8% (6.6 – 13.0)
							8 years: 8.1% (5.4 – 10.8)
							9 years: 7.3% ( 3.7 – 10.9)
							10 years: 4.1% (2.1 – 6.1)
							11 years: 3.2% (1.9 – 4.5)
							12 years: 3.2% (1.6 – 4.8)
							13 years: 2.4% (0.9 – 3.9)
							14 years: 0.0
							15 years: 0.0
Fotouhi (2007) [9]	3673	7-15 years	≥ + 2.00 D	96.8	16.6	13.6 – 19.7	7 years: 28.9% (22.6 – 35.2)
Dezful, Iran			RESC				8 years: 22.7% (16.4 – 28.9)
			ca				9 years: 16.7% (12.0 – 21.4)
							10 years: 12.4% (7.9 – 17.0)
							11 years: 12.9% ( 8.3 – 17.5)
							12 years: 16.9% (12.3 – 21.5)
							13 years: 14.1% (10.6 – 17.6)
							14 years: 13.0% (9.8 – 16.1)
							15 years: 10.3% (1.5 – 19.1)
Hashemi (2010) [24]	345	5-10 years	≥ + 2.00 D	Not stated	10	Not available	Not available
Tehran, Iran			Right eye				
			ca				
Ostadimoghaddam (2011) [25] Mashhad, Iran	639	5-15 years	≥ + 2.00 D	Not stated	19.05	15.7 – 22.4	Not available
			At last one eye was hyperopic				
			ca				
Rezvan (2012) [26]	1551	6-17 years	≥ + 2.00 D	76.8	5.4	4.3 – 6.5	8 years: 6.8% (2.7–11.0)
Bojnourd, Iran			RESC				9 years 8.2% (3.9–12.5)
			ca				10 years: 8.3% (4.1–12.6)
							11 years: 5.6 % (2.0–9.2)
							12 years: 3.8% (1.3–6.2)
							13 years: 2.3% (0.3–4.3)
							14 years: 2.5% (0.3–4.6)
Yekta (2010) [27]	2130	7-15 years	≥ + 2.00 D	87.88	5.04	3.5 – 6.6	7 years: 8.9% (6.1 – 11.8)
Shiraz, Iran			RESC				8 years: 7.7% (1.9 – 13.5)
			ca				9 years: 4.8% (1.6 – 8.1)
							10 years: 7.0% (2.8 – 11.1)
							11 years: 2.1% (0.7 – 5.8)
							12 years: 3.0% (1.2 – 4.8)
							13 years: 2.2% (0.6 – 3.8)
							14 years: 5.9% (0.1 – 11.8)
							15 years: 0.0
Robaei (2005) [28]	1765	6 years	≥ + 2.00 D	Not stated	9.8	Not available	-
			Right eye				
			ca				
SMS, Sydney, Australia							
Ip (2008) [29]	4094	6 years	≥ + 2.00 D	Not stated	-	-	6 years: 13.0% (9.1 – 16.8)
SMS, Sydney, Australia		12 years	Eye with greater refractive error				12 years: 5.0% (3.5 – 6.5)
			ca				
Ip (2008) [30]	2353	11-15 years	≥ + 2.00 D	Not stated	3.5	2.8 – 4.1	Not available
SMS, Sydney, Australia			Both eyes				
			ca				
Robaei (2006) [31]	2353	12 years	≥ + 2.00 D	75.3	5	Not available	Not available
			Both eyes				
			ca				
SMS, Sydney, Australia							
Grönlund (2006) [32]	143	4-15 years	≥ + 2.00 D	Not stated	9.1md	Not available	Not available
			At last one eye was hyperopic				
Gothenburg, Sweden			ca				
Laatikainen (1980) [33] Uusimaa County, Finland	822	7-15 years	≥ + 2.00 D	Not stated	9.7	Not available	7 – 8 years: 19.1% (13.0 – 25.1)
			Right eye				
			cr				9 – 10 years: 6.9% (3.5 – 10.3)
							11 – 12 years: 11.7% (7.5 – 15.9)
							14 – 15 years: 3.6% (1.1 – 6.1)
O’Donoghue (2012) [34] Northern Ireland (NICER)	1053	6-7 years	≥ +2.00D	62.0 in children 6–7 years 65.0 in children 12–13 years	26	20 – 33	6-7 years: 26% (20–33)
					14,7	9.9 – 19.4	12-13 years: 14.7% (9.9 – 19.4)
		12-13 years	RESC				
			ca				
Logan (2011) [35] Birmingham, England (AES)	596	6-7 years	≥ + 2.00 D	Not stated	12.3	8.8–15.7	Not available
					5.4	2.8 – 8.0	
		12-13 years	Either/both eyes				
			ca				
Naidoo (2003) [36]	4890	5-15 years	≥ + 2.00 D	87.3	2.6	Not available	5 years: 2.7% (0.6 – 4.8)
			RESC				
			ca				
Durban area, South Africa							6 years: 2.4% (0.7 – 4.1)
							7 years: 2.8% (0.9 – 4.7)
							8 years: 1.3% (0.1 – 2.6)
							9 years 2.9% (0.1 – 5.7)
							10 years: 3.4% (1.8 – 4.9)
							11 years: 3.5% (1.9 – 5.1)
							12 years: 3.2% (1.2 – 5.1)
							13 years: 2.9% (0.3 – 5.5)
							14 years: 1.9% (0.6 – 3.2)
							15 years: 0.7% (0.0 – 1.8)
Maul (2000) [37]	5303	5-15 years	≥ + 2.00 D	75.8	19.3	Not available	Males:
			RESC				5 years: 22.7% (18.0 – 27.4)
			ca				15 years: 7.1% (3.5 – 10.6)
							Females:
							5years: 26.3% (22.0 – 30.6)
							15 years: 8.9% (3.7 – 14.1)
La Florida, Chile							
Czepita (2008) [38]	Urban: 1200	10-14 years	≥ + 1.50 D	Not stated	Urban: 7.1	5.6 – 8.5	Urban (N = 1200):
			Right eye		Rural: 30.8	27.9 – 33.7	10 years: 8.3% (5.2 – 11.3)
			cr				11 years: 4.1% (1.6 – 6.6)
							12 years: 9.9% (5.8 – 14.0)
							13 years: 7.7% (4.3 – 11.1)
							14 years:5.3% (2.2 – 8.3)
							Rural (n = 1006)
							10 years: 33.3% (27.1 – 39.5)
Czeczecin, Poland	Rural:1006						11 years: 28.4% (22.1 – 34.7)
							12 years: 26.9% (20.9 – 32.9)
							13 years: 30.5% (24.4 – 36.5)
							14 years:36.4% (28.7 – 44.1)
Kleinstein (2003) [39] CLEERE Study, USA	2523	5-17 years	≥ + 1.25 D in each meridian	Not stated	12.8	11.5 – 14.1	Not available
			Right eye				
			ca				
Zadnik (2003) [40]	2583	7-12 years	≥ + 1.25 D^§^	Not stated	8.6	Not available	Not available
			Right eye				
			ca				
CLEERE Study, USA							
Dandona (1999) [41]	599	0-15 years	≥ + 1.00 D	Not stated	41.14	24.9 – 58.0	Not available
			Eye with higher refractive error				
Andhra Pradesh, India							
			cr				
Shrestha (2011) [42]	2236	5-16 years	≥ + 1.00 D^†^	Not stated	20,3	Not available	Not available
			Either/both eyes				
			cr				
Jhapa, Nepal							
Czepita (2007) [43]	4422	6-18 years	≥ + 1.00 D	Not stated	13.05	Not available	6 years: 36.5% (31.8 – 41.3)
							7 years: 19.2% (15.4 – 22.9)
							8 years: 17.4% (13.8 – 21.0)
							9 years 11.3% (8.3 – 14.3)
							10 years: 11.0% (8.0 – 14.0)
							11 years: 10.9% (8.0 – 14.0)
							12 years: 8.3% (5.6 – 10.9)
							13 years: 11.8% (8.1 – 15.5)
							14 years: 8.2% (5.3 – 11.2)
							15 years: 8.6% (5.4 – 11.8)
							16 years: 2.8% (0.6 – 5.1)
							17 years: 2.5% (0.3 – 4.7)
							18 years: 3.2% (0.7 – 5.7)
			Right eye				
			cr				
Szczecin, Poland							
Vilareal (2003) [44]	1035	12-13 years	≥ + 1.00 D	Not stated	6	Not available	Not available
			ca				
Monterrey, Mexico							
Vilareal (2000) [45]	1045	12-13 years	≥ + 1.00 D	Not stated	8.4%	Not available	Not available
			Right eye				
			cr				
Götemborg area Sweden							
Hashemi (2004) [46]	412	5-15 years	≥ + 0,50 D	Not stated	78.6	74.6 – 82.6	Not available
			Right eye				
			ca				
Tehran, Iran							
Dandona (2002) [47]	2603	0-15 years	≥ + 0,50 D	Not stated	62.6	57.0 – 68.1	Not available
			Eye with higher refractive error				
Andhra Pradesh, India							
			cr				
Niroula (2009) [48]	964	10-19 years	≥ + 0,50 D^‡^	Not stated	1.24	Not available	Not available
			Both eyes				
			cr				
Pokhara, Nepal							

y = years (age); CI: Confidence Interval; SE: mean spherical equivalent; RESC: The Refractive Error Study in Children; ca: cycloplegic autorefraction; cr: cycloplegic retinoscopy.

† study did not mention SE in its definition of hyperopia.

‡ It was considered +0,5 diopter or more spherical power.

§ Define as +1.25 D or more in both meridians.

The meta-analysis was performed using a Microsoft Excel spreadsheet [49]. Differences in the populations studied, especially ethnicity, have a non-random impact on prevalence. The random effects model was therefore used in order to obtain the effect summary and its confidence interval. The adequacy of the effect summary depends on the homogeneity assumption. Heterogeneity was measured using the Q test and was quantified using I^2^ statistics. Heterogeneity tests having a p-value <0.1 were considered statistically significant.

This systematic review was performed according to the PRISMA [50] and MOOSE [51] Statements. The study was approved by the Federal University of Pelotas School of Medicine Research Ethics Committee and follows the Declaration of Helsinki guidelines [52].

## Results

### Hyperopia prevalence by age in children

The review included 40 cross-sectional studies on prevalence and/or assessment of risk factors for hyperopia. Eighteen studies were conducted in Asia, of which six were carried out in China and five in India. The other Asian countries were: Nepal (three studies), Malaysia (two studies), Cambodia and the Democratic Republic of Laos (one study each). Seven studies are from Europe (two were conducted in the United Kingdom; Poland and Sweden carried out two studies each and one study was conducted in Finland). Six studies are from the Middle East (Iran). Four studies were conducted in Australia, two in the United States and one study each in South Africa, Chile and Mexico.

All samples of children used in the studies were population-based or school-based, except the study that used a sample of children from a private school in Xiamen, China [13].

In most studies included in this review, the cut-off point for hyperopia was based on the Refractive Error Study in Children (RESC) protocol used in multicenter studies [53]. Spherical equivalent refraction (SE) for hyperopia was ≥ +2.00D (one or both eyes, if none the eyes are myopic). The studies used data from one or both eyes to determine prevalence. However, some studies used different cut-off points [38–48, 54], thus underestimating or overestimating hyperopia prevalence compared to studies using the RESC protocol. Some studies performed the examination on the right eye only, thereby underestimating the prevalence of hyperopia [38, 43].

**Figure 2:**
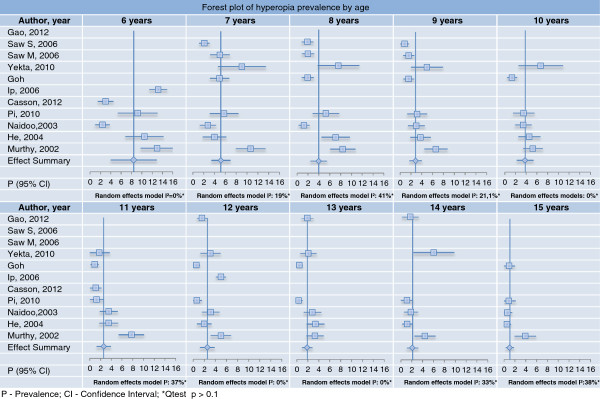
**Forest plot of hyperopia prevalence by age.**

The meta-analysis indicates that hyperopia prevalence decreases as age increases, with a summary prevalence measure of 5% at age 7, 2-3% between age 9 and 14 and around 1% at age 15. Various studies of children aged 6 to 8 presented large confidence intervals. I^2^ indicates homogeneity among the studies regarding specific age (Figure 2).

In studies using the 5-15 age group and ≥ +2.00 D (RESC) cut-off, hyperopia prevalence ranged from 2.1% [18] to 19.3% [25, 37] (Table 1).

Although there is literature indicating a direct association between AL and age, only a few studies have assessed its distribution by specific ages [40, 55].

### Gender and hyperopia in children

Most studies showed no statistically significant association between gender and hyperopia (Table 2) [9, 11, 14, 17, 19, 20, 23, 25–27, 30, 32, 34, 36, 39–41, 46–48, 56, 57]. With regard to ocular components, on average girls appear to have shorter AL when compared to boys [3, 30, 55, 58].

**Table 2:** **Hyperopia associated factors**

Author (Year)	Location	Hyperopia associated factors
Ip (2008) [29]	Sydney Myopia Study (SMS)	**GENDER:** Age 6, girls were more hyperopic 15.5% (95%CI 12.7 – 18.4) than boys 10.9% (95%CI 8.5 – 13.2) (p = 0.005). Age 12, boys: 5.1% (95%CI 3.8–6.5), girls: 4.7% (95%CI 3.5–6.0), NS.
	Australia	
		**ETHNICITY:** At age 6, more prevalent in European Caucasian 15.7% (95%CI 13.2–18.2) when compared with East Asian 6.8% (95%CI 4.0–9.5) and South Asian 2.5% (95%CI 0.0–7.5). East Asian, South Asian and Middle Eastern 8.4% (95%CI 1.6–15.2) do not present differences among their prevalence. At age 12, more prevalent in European Caucasian, 6.4% (95%CI 5.2–7.7) than East Asian 2.0% (95%CI 1.0–3.0). No difference between East Asian and Middle Eastern 7.4% (95%CI 2.7–12.0) and European Caucasian and Middle Eastern.
		**PARENTAL EDUCATION:** Age 12, Maternal Education, (p = 0.055).
		**SOCIO-ECONOMIC STATUS:** Age 6, Maternal Occupation, (p = 0.02). Home Ownership or Paternal Education or Employment (p > 0.1), after adjusted for demographic factors (gender, ethnicity, parental education, parental employment). Parental Employment was associated with moderate hyperopia (≥ + 2.00 D), (p = 0.02).
Ip (2008) [30]	Sydney Myopia Study (SMS)	**GENDER:** Age 11–15, no difference among boys 3.6% (95%CI 2.6–4.7) and girls 3.3% (95% CI 2.2–4.4). Age 12, girls showed a lower mean spherical equivalent (SE) (+0.39D) than boys (+0.58D), (p = 0.04).
	Australia	
		**ETHNICITY:** European Caucasian 4.4% (95%CI 3.6–5.3) are more likely to have moderate hyperopia (≥ + 2.00 D) than East Asian 1.1% (95%CI 0.2–2.1), South Asian 0.0%(-) and other mixed ethnicity 1.7% (95%CI 0.0–3.6). Middle Eastern 6.1% (95%CI 1.5–10.7) are more likely to have moderate hyperopia than South Asian. There was no difference between European Caucasian and Middle Eastern. Age 12, Middle East showed a lower mean of SE (+0.71) than Caucasian (+0.82D) (p = 0.03). Caucasian had the highest mean SE (+0.82D) when compared to all ethnicities together (+0.04D), (p < 0.0001).
Ip (2008) [59]	Sydney, Australia	**OUTDOOR ACTIVITIES:** Age 12, greater time, (β coefficient = 0.03, p <0.0001), and weakly correlated with near-work activities (r =0.1, p < 0.0001).
		**NEAR WORK ACTIVITIES:** Parental Reports of Close Reading Distance (<30 cm) (p < 0.0001), after adjustment for age, sex, ethnicity, and school type.
Rose (2008) [8]	Sydney Myopia Study (SMS)	**OUTDOOR ACTIVITIES:** Age 6 and 12, Greater Number of Hours, *p* = 0.009 and *p =* 0.0003 respectively, after adjustment for gender, ethnicity, parental myopia, near work, maternal and parental education, and maternal employment.
	Australia	
		**NEAR WORK ACTIVITIES:** Age 12, Greater Levels of Near-work Activity, p =0.8.
Maul (2000) [37]	La Florida, Chile	**AGE:** 5**–**15, inverse relation (p < 0.05).
		**GENDER:** Age 5–15, girls OR = 1.21 (95% CI 1.03-1.43).
Zhao (2000) [10]	Shunyi, China	**AGE:** 5–15, inverse relation OR = 0.75 (95% CI 0.71-0.79).
		**GENDER:** Age 5–15, girls OR = 1.51 (95%CI 1.08-2.13).
Zhan (2000) [13]	Xiamen city, Xiamen Countryside and Singapore, China	**RESIDENCE AREA:** Age 6–7, Residence Zone, p = 0.50.
He (2004) [11]	Guangzhou, China	**AGE:** 5–15, inverse relation OR = 0.77 (95% CI 0.73-0.81).
		**GENDER:** Age 5–15, NS p = 0.233.
		**PARENTAL EDUCATION:** inverse relation OR = 0.81 (95%CI 0.66-0.98).
Pi (2010) [14]	Yong Chuan District, Western China	**AGE:** 6 – 15, inverse relation OR = 0.831 (95%CI 0.728-0.948), p < 0.01.
		**GENDER:** Age 6–15, χ^2^ = 2.977, NS p = 0.08.
Dandona (2002) [47]	Andhra Pradesh, India	**AGE:** 0 – 5, were more hyperopic than those 10 – 15, OR = 3.34 (95%CI 2.69–4.14), p < 0.05. and 6 – 9 were more hyperopic than 10 – 15, OR = 1.72 (95%CI 1.41–2.10), p < 0.05
		**GENDER:** Age 0–15 OR:1.19 (95%CI 0.76 – 1.86), NS.
		**SOCIO-ECONOMIC STATUS:** Base Group: extreme lower income, Upper OR = 2.27% (95%CI 0.59 – 8.77), Middle OR = 2.21% (95%CI 0.89 – 5.50), Lower OR = 1.76% (95%CI 0.74 – 4.19). **RESIDENCE AREA:** Two Rural Areas, OR = 2.84 (95%CI 2.16-3.75) and OR = 1.50 (95%CI 1.17-1.92) when compared with Urban.
Laatikainen (1980) [33]	Uusimaa County, Finland	**AGE:** 7–15 years, inverse relation, x^2^ = 28.617, p < 0.0005.
Grönlund (2006) [32]	Gothenburg, Sweden	**AGE:** 4 – 15, Correlation SE OD: r = -0.37, p < 0.0001 and SE OS: R = -0.33, p < 0.0001.
		**GENDER:** Age 4–15, SE OD (p = 0.61) and SE OS: (p = 0.85).
		**OBS:** The mean and standard deviation (SD) of the spherical equivalent (SE) was used in this study.
Dandona (2002) [22]	Andhra Pradesh, India	**AGE**: 7–15, NS.
		**GENDER:** Age 7–15, NS.
		**PARENTAL EDUCATION:** Education of the father (grade level achievement: none, 1–5, 6–12, 13–15, 15 or more), NS.
		**SOCIO-ECONOMIC STATUS:** Extreme Lower, Lower, Middle, Upper, NS.
Dandona (1999) [41]	Andhra Pradesh, India	**AGE**: 0 – 15, NS.
		**GENDER:** Age 0–15, NS.
		**SOCIO-ECONOMIC STATUS:** Extreme Lower, Lower, Middle, Upper, NS.
Murthy (2002) [21]	New Delhi, India	**GENDER:** Age 11–13, girls OR = 1.72 (95% CI 1.05-2.81).
		**PARENTAL EDUCATION:** Age 11–13, Child Education, inversely associated OR = 0.89 (95%CI 0.81-0.99).
Hashemi (2004) [46]	Tehran, Iran	**AGE** 5–15, inverse association, S p < 0.001.
		**GENDER:** Age 5–15, Boys, 78.6% (95%CI 74.6 – 82.6), Girls, 73.2 (95%CI 68.5 – 77.9), NS.
Fotouhi (2007) [9]	Dezful, Iran	**AGE** 7–15, inverse relation OR = 1.73 (95%CI 0.83-0.94), p < 0.001.
		**GENDER:** Age 7–15, boys 16.1% (95% CI 11.0–21.1), girls 16.1% (95%CI 11.0–21.1), NS.
		**RESIDENCE AREA:** Rural, OR = 2.0 (95%CI 1.09-3.65).
Yekta (2010) [27]	Shiraz, Iran	**AGE:** 7–15, inverse relation OR = 0.84 (95%CI 0.73-0.97), S, p = 0.021.
		**GENDER:** Age 7–15, boys: 5.17% (95%CI 3.19–7.15), girls, 4.90% (95%CI 2.32–7.48), NS, p = 0.863.
Ostadimoghaddam (2011) [25]	Mashhad, Iran	**AGE:** 5 – 15 inverse relation, S, (p < 0.001).
		**GENDER:** Age 5–15, NS, p = 0.724.
Goh (2005) [17]	Gombak District, Malaysia	**AGE:** 7–15, inverse relation OR = 0.72 (95%CI 0.62-0.82).
		**GENDER:** Age 7–15, boys, 1.7% (95%CI 1.1–2.3), girls, 1.4% (95%CI 0.8–2.1).
		**ETHNICITY:** Age 7–15, “other” ethnicities were more hyperopic OR = 3.72 (95%CI 1.34-10.35) than Malaysian and Chinese. No differences were found among Malaysian 1.5% (95%CI 1.1–1.9), Chinese 1.1% (95%CI 0.4–1.7) or Indian 2.0% (95%CI 0.1–3.9).
		**PARENTAL EDUCATION:** Parental with highest level of schooling, NS.
Varma (2009) [56]	Multi-Ethnic Pediatric Eye Disease Study Group (MEPEDS)	**AGE:** 6 – 72 months, Hispanic children, inverse relation, (6–11 months) vs (60–72 months) OR = 1.46 (95%CI 1.08–1.98) (P = 0.0017). Age 6–72 months, African-American, NS.
	Los Angeles County, California USA	**ETHNICITY:** Age 6–72 months, Hispanic were more hyperopic 27.1% (95%CI 24.0 – 30.1) than African-American 21.1% (95%CI 17.9 – 24.3), after controlling for age, S, p < 0.001. Age 6–11 months and 36–47 months Hispanic are more hyperopic 35.1% (95%CI 29.7 – 40.5) and 29.9% (95%CI 26.0 – 33.8) than African-American, 18.1% (95%CI 13.5 – 22.7) and 20.7% (95%CI 17.3 – 24.1) respectively.
Pokharel (2000) [18]	Mechi Zone, Nepal	**AGE:** 5 – 15, as continuous variable, NS.
		**GENDER:** Age 5–15, girls OR = 1.44 (95%CI 1.02-2.03).
Czepita (2007) [43]	Czeczecin, Poland	**AGE** 6–18, negative correlation, Sr = 0.907, S, p < 0.001
		**GENDER**: Age 6–18, boys 40.3%(95% CI 38.5 – 42.1) are more hyperopic than girls, 35.3% (95%CI 33.6 – 37.0).
Naidoo (2003) [36]	Durban area, South Africa	**AGE:** 5 – 15 years, NS.
		**GENDER:** Age 5–15, NS.
		**PARENTAL EDUCATION:** parent with the highest education (grade level achievement: none, 1–5, 6–12, 13–15, 15 or more), NS.
Garner (1990) [60]	Island of Efaté, Republic of Vanatu, Melanesia	**AGE:** 6 – 17, age groups Melanesian, NS.
	Kuala Lumpur, Malaysia	**ETHNICITY:** Age 6, Malaysian were more hyperopic than Melanesian.
Kleinstein (2003) [39]	Collaborative Longitudinal Evaluation of Ethnicity and Refractive Error Study Group	**ETHNICITY**: Age 5 – 17, white are more hyperopic 19.3% (95%CI 16.9 – 21.7) than Asians 6.3% (95%CI 4.1 – 8.4) and African-Americans 6.4% (95%CI 4.3 – 8.5), x^2^ = 236.15, S, p < 0.001. Age 5–17 white didn’t differ from Hispanics 12.7% (95% CI 9.7 – 15.7), NS, p = 0.48. Age 5–17 Asians and Africa-Americans, NS, p = 0.07.
	(CLEERE) Study	
	Eutaw, Alabama; Irvine, California and Houston, Texas USA	
		**GENDER:** Age 5–17, boys 12.6% (95%CI 10.8 – 14.4) are more hyperopic than girls 13.1% (95%CI 11.2 – 15.0).
Zadnik (2003) [40]	Collaborative Longitudinal Evaluation of Ethnicity and Refractive Error Study Group	**AGE:** Age 6 to 7 and age 8 were more hyperopic than 9 to14, S, p < 0.0001.
	(CLEERE) Study	
	Eutaw, Alabama; Irvine and Orinda, California and Houston, Texas USA	
Giordano (2009) [54]	Baltimore Pediatric Eye Disease Study (BPEDS)	**ETHNICITY:** 6 – 72 months, white are more hyperopic (≥ + 1.00) than African-American OR = 1.62 (95%CI 1.51-1.74). White, 6 – 11: 33.0% (95%CI 22.9 – 43.1), 12 – 23: 30.3% (95%CI 23.5 – 37.1), 36 – 47: 27.5% (95%CI 21.5 – 33.5), 48 – 59: 33.3% (95%CI 26.8 – 39.9) and 60 – 72: 31.5% (95%CI 24.5 – 38.4) months are more hyperopic (≥ + 2.00D) than African American at same age ranges, 21.2% (95%CI 12.4 – 30.0, 15.7% (95%CI 10.5 – 20.9), 16.2% (95%CI 11.5 – 20.9), 17.2% (95%CI 12.6 – 21.8) and 17.4% (95%CI 12.6 – 22.1) respectively.
	USA	
Borchert (2011) [61]	Baltimore Pediatric Eye Disease Study (BPEDS)	**AGE:** 6 – 72 months. Those 12 – 23 months and 24 – 35 months are more hyperopic than 60 – 72 months OR = 0.81(95%CI 0.68 – 0.97) and OR = 0.74 (95%CI 0.62 – 0.88) respectively.
	USA	**ETHNICITY:** Age 6–72 months, Non-Hispanic white, children are more hyperopic than African-American OR = 1.63 (95%CI 1.43 – 1.87). Age 6–72 months, Hispanic white are more hyperopic than African-American OR = 1.49 (95%CI 1.32 – 1.68).
		**SOCIO-ECONOMIC STATUS:** Age 6–72 months with Health insurance, OR = 1.51 (95%CI 1.12 – 1.69).
O’Donoghue (2012) [34]	Northern Ireland Childhood Errors of Refraction	**AGE:** 6 – 7 are more hyperopic 26% (95%CI 20–33) than 12 – 13 years, 14.7% (95%CI 9.9 - 19.4), p < 0.005.
	(NICER)	**GENDER:** Age 6–7, NS. Age 13–13, S.
	Northern Ireland	
Dirani (2010) [57]	The Strabismus, Amlyopia and Refractive Errors in Singaporean children	**AGE:** 6 – 72 months, inverse relation, Age 6 – 11.9 months 15.7% (95%CI 10.6 – 22.2), Age 24 – 35.9 months 6.8% (95%CI 4.6 – 9.6), Age 36 – 47.9 months 5.1% (95%CI 3.3 – 7.3) and age 60 – 72 months 5.7% (95% CI 3.8 – 8.0), S, p _trend_ = 0.001.
	(STARS)	
	Singapura	**GENDER:** Age 6–72 months, boys 6.6% (95%CI 5.1 – 7.7), girls: 9.4% (95%CI 7.9 – 11.1), NS, p = 0.75.
Casson (2012) [20]	Vientiane Province, Lao PDR	**GENDER:** 6 – 11, NS, p = 0.95.
Uzma (2009) [23]	Hyderabad, Índia	**GENDER:** 7 – 15, Urban, boys 1.5% (95%CI 0.7–2.3), girls, 1.4% (95%CI 0.6–2.2). Rural, boys, 2.7% (95%CI 1.3–4.1), girls, 2.1% (95%CI 0.9–3.3), NS.
		**RESIDENCE AREA:** Age 8, 9, 12 and 13, Rural, are more hyperopic than urban, 8.1% (95%CI 5.4–10.8) v 2.0% (95%CI 0.4–3.6), 7.3% (95%CI 3.7–10.9) v 1.7% (95%CI 0.8–2.6), 3.2% (95%CI 1.6–4.8) v 0.4% (95%CI 0.0–0.8) and 2.4% (95%CI 0.9–3.9) v 0.2% (95%CI 0.0–0.4), respectively.
Rezvan (2012) [26]	Bojnourd, Iran	**AGE:** 6 – 17, inverse relation, S, p < 0.0001.
		**GENDER:** Age 6–17, boys, 4.4% (95%CI 2.8–5.9), girls, 6.1% (95%CI 4.5–7.7), NS.
Saw (2006) [16]	Gombak District, Kuala Lumpur Malaysia Singapore	**AGE:** 7, Malaysian are more hyperopic (5%) than Singapore (2.1%), Prevalence difference, -22.9% (95%CI -24.8 to -20.9), S, p < 0.001.
		**GENDER:** Age 7–9, Malaysian boys are more hyperopic (3.2%) than Singaporean boys (1.3%), Prevalence difference, -21.9% (95%CI -23.3 to -20.6), p < 0.001.
		**ETHNICITY:** Age 7–9, Singaporean, are less hyperopic (1.7%) than Malaysian (2.9%), Prevalence difference, -21.1% (95%CI -22.1 to -20.2), p = 0.005.
		**PARENTAL EDUCATION:** Age 7–9, Completed Education Level of the Father, NS.
		**OBS:** Differences in the prevalence rates of hyperopia between Malaysia and Singapore were considered significant if the 95% confidence intervals of the differences in the prevalence rates did not cross zero and p values were <0.05.
Logan (2011) [35]	Birmingham, England (AES)	**ETHNICITY:** Age 6–7, White European are more hyperopic, 22.9% (95%CI 12.9% – 32.8%) than South Asian 10.3% (95%CI 6.2% - 14.4%) and Black African Caribbean 9.1% (95%CI 0.5 – 17.7). South Asian v Black African Caribbean, NS. Age 12 – 13, White European 10.4% (95%CI 4.8% – 16.1%) v South Asian 2.6% (95%CI 0.0 - 5.6%), NS.
Czepita (2008) [38]	Szeczecin, Poland	**RESIDENCE AREA:** Age 6**–**18, living in the city, are less hyperopic than those in the countryside, S, p < 0.001.
Gao (2012) [19]	Phnom Penhn, Cambodia	**AGE:** 12, 13 and 14, Prevalence Rates, 0.7% (95%CI 0.4–1.0), 0.7% (95%CI 0.4–0.9) and 0.8% (95%CI 0.3–1.3) respectively, NS.
		**GENDER:** Age 12–14, boys: 0.6% (95%CI 0.3–0.8), girls, 0.9% (95%CI 0.6–1.1), NS.
		**RESIDENCE AREA:** Age 12–14, urban, 1.4% (95%CI 0.1–1.7) v rural, 0.4% (95%CI 0.2–0.6), NS.
Niroula (2009) [48]	Pokhara, Nepal	**GENDER:** 10 – 19, boys, 1.48% (95%CI 0.3–2.6), girls, 1.02% (95%CI 0.1–1.9), NS.

OR: odds ratio; CI: confidence interval; SE: spherical equivalence; NS: non-significant; S: significant.

According to some studies however, girls are more likely to be hyperopic when compared to boys. In Australia, girls aged 6 are more likely to be hyperopic (15.5%) (95% CI 12.7-18.4) than boys of the same age (10.9%) (95% CI 8.5-13.2) (p = 0.005), although this difference was not found among children aged 12 in the same study [29]. Similarly, studies conducted in Chile, China and Nepal with children aged 5-15 years showed that girls are more likely to be hyperopic than boys: OR = 1.21 (95% CI 1.03-1.43) [37], OR = 1.51 (95% CI 1.08-2.13) [10] and OR = 1.44 (95% CI 1.02-2.03), [18] respectively. However, in a study conducted in Poland boys aged 6-18 years showed higher hyperopia prevalence (40.3%) (95% CI 38.5-42.1) when compared to girls in the same age range (35.3%) (95% CI 33.6 - 37.0) [43].

### Ethnicity and hyperopia in children

Some studies have shown that there is no significant difference in hyperopia prevalence between Caucasian and Hispanic children [39] or between Caucasian and Middle East children [29, 30]. There is also evidence that Caucasian children are more hyperopic than African-American [39, 54, 56, 61], Black [35] and Asian (East and South Asia) children [29, 30, 35]. With regard to specific ethnic groups, there is no difference between hyperopia prevalence among Malay, Chinese and Indian children [17], although Malaysian children are more hyperopic than Singaporean (p = 0.005) [16] and Melanesian children [60]. It was also found that children of other ethnicities (not specified) are more likely to be hyperopic than Melanesian children OR = 3.72 (95%CI 1.34-10.3) [17] (Table 2).

In the South African study, hyperopia prevalence among children aged 7 years was only 2.8% [36]. The majority of the South African population is Black, followed by Asians (9.4%) and Caucasians (6.6%). In the Malay study, hyperopia prevalence among children aged 10 years was 1.4% [17]. The ethnic composition of the region is mostly Malay but approximately 28% of individuals have Chinese origin. The lowest hyperopia prevalence (0.5%) was found in a study in Guangzhou, one of the most developed cities in southern China [11].

Regarding ocular components in different ethnicities, on average it was found that AL is shorter and CC is flatter among Caucasian children [3, 30, 62].

### Parental education and socio-economic status and hyperopia in children

Most of the reviewed studies showed no significant association between parental education and hyperopia in children (Table 2) [16, 17, 21, 22, 27, 36, 47]. In an Australian study, although there was no significant association between paternal education and hyperopia among children under 6 years of age, maternal education showed an inverse association with the presence of hyperopia among children aged 12 (p = 0.055) [29]. In a Chinese study the high level of parental education was a protective factor against the presence of hyperopia among children aged 5-15 years, OR = 0.81 (95% CI 0.73 - 0.81) [11].

Regarding socio-economic status, maternal employment is directly related to hyperopia in 6-year-old children in Australia (p = 0.02), although it is not associated with family income or paternal employment (p > 0.1) [29]. In the same study, an association between both parents being employed and hyperopia ≥ +2.00 D was found among 6-year-old children, after adjusting for gender, ethnicity and parental education (p = 0.02) [29].

Each of the three Indian studies with children aged 0-15 years had different cut-offs for hyperopia (≥ + 2.00D, ≥ + 1.00D and ≥ +0.5 D) but none of them showed association between socio-economic status (classified according to family income) and hyperopia [22, 41, 47].

In a study conducted in the United States, children aged 6-72 months with health insurance coverage showed a greater chance of having hyperopia when compared to those with no health insurance, OR = 1.51 (95% CI 1.12 - 1.69) [61].

### Area of residence and hyperopia in children

There are few studies on the association between area of residence (urban or rural) and hyperopia prevalence in children. In an Indian study, children aged 0-15 years who lived in two rural areas were more likely to be hyperopic when compared to those living in urban areas, OR = 2.84 (95% CI 2.16-3.75) and OR = 1.50 (95% CI 1.17-1.92) respectively (Table 2) [47]. In another study conducted in India with children aged 7-15 years, those aged 8, 9, 12 and 13 years living in rural areas presented higher prevalence of hyperopia than those of the same age living in urban areas (Table 2) [23].

An Iranian study showed that children aged 7-15 years living in rural areas are more likely to be hyperopic than those living in urban areas, OR = 2.0 (95% CI 1.09-3.65) [9] and another study in Poland reported that children aged 6-18 years living in urban areas showed lower frequency of hyperopia when compared to children living in rural areas (p < 0.001) (Table 2) [38].

Two reviewed articles (one conducted in China with children aged 6-7 years and the other in Cambodia with children aged 12-14 years) showed no significant association between area of residence and hyperopia [13, 19] In the Cambodian study, hyperopia prevalence rates among children living in urban and rural areas were 1.4% (95% CI 0.1 - 1.7) and 0.4% (95% CI 0.1 - 1.9) respectively (Table 2) [19].

### Outdoor activities and hyperopia in children

Rose et al. noted that children aged 6 and 12 years in Australia who spent more time per week doing outdoor activities (outdoor sports, picnics and walking) were more hyperopic than those who spent less time practicing these activities, adjusted for gender, ethnicity, presence of myopia in parents, near activities, and maternal and paternal education and working mothers (p = 0.009 and p = 0.0003, respectively) (Table 2) [8]. These authors also noted that there was a statistically significant trend toward greater hyperopic spherical equivalent refraction as tertiles of outdoor activities increased and tertiles of near activities decreased [8]. In the same study, Rose concluded that hyperopic spherical equivalent refraction was more common in children who dedicated less time to near activities and more time to outdoor activities [8].

Spending time engaged in outdoor activities was slightly associated with hyperopia (β = 0.03, p < 0.0001) among 12-year-old children in Australia. That study found that children who performed near activities (reported by parents), such as reading distance (<30cm), were significantly associated with less hyperopia (p < 0.0001), after adjusting for age, gender, ethnicity and type of school (Table 2) [59].

In the United States, Mutti et al. examined 366 children with mean age of 13.7 ± 0.5 years and showed (using the Wilcoxon rank-sum test) that myopic children spend more time reading for pleasure (p = 0.034) and less time playing sports (p = 0.049) compared with hyperopic children [7].

## Discussion

There are several studies on hyperopia prevalence in childhood, but a great difficulty arises when attempting to compare them. This is partly due to the methodological characteristics of each study. Regarding the diopter value, there is no consensus on the cut-off point for diagnosing children as hyperopic, nor on what is the most appropriate measure: a greater, or lesser, hyperopic corneal meridian or spherical equivalent refraction [2]. However, cycloplegia followed by retinoscopy or autorefraction is the acceptable way of testing to diagnose ametropias, although doubts remain as to its accuracy in children with darker irises [63]. Most studies classify an individual as being hyperopic after binocular examination, but others use the eyes separately as unit samples or examine only one of the eyes (usually the right eye) relying on evidence of good correlation between ametropia in both eyes [2].

The RESC protocol has been used as a way of standardizing the methodology applied in studies on refractive errors, thus improving the comparability of results between child populations [53]. Hyperopia has an inverse association with age, is more common in Caucasian children and in those who live in rural areas or spend more time doing outdoor activities and it shows inconsistent results regarding association with gender, socio-economic status and parental education.

There is consistency among the studies about the inverse association between hyperopia and age. Although there are studies stating that slow growth in AL lasts until around the age of 12-14 years [5, 55, 64], emmetropization is minimal after the age of three, [6] and does not explain the decrease in hyperopia by age after 5 years-old.

Studies included in the meta-analysis were selected due to their methodological similarity and high response rate. The larger confidence intervals among those aged 6 to 8 indicate a less precise estimate of prevalence which is related to smaller sample size in these specific ages. However, it might also reflect greater difficulty in performing examinations in younger children, or greater variability in different populations in this age range, such as the heredity of refractive error or ocular characteristics of components such as axial length among different ethnicities.

The conflicting results when assessing the association between gender and hyperopia may be related to gender representativeness in the studies. On the one hand, the gender ratio is fairly even, suggesting good representativeness. Yet in some cultures girls have more difficulty in accessing schools, which could imply selection bias in hyperopia prevalence. On the other hand, females have greater acceptance and participation in studies, trials and interviews with scientific purposes which in turn could lead to positive selection bias [25].

The particularly low hyperopia prevalence could be partly explained by ethnicity, such as in Durban, South Africa [36], where the majority of the population are Black, followed by Asians. Regarding ocular components, axial length in both Africans and Asians is longer than in Caucasian individuals.

Literature shows that populations with high myopia prevalence rates generally have low hyperopia prevalence, as in China [11, 30]. This aspect may influence the prevalence of hyperopia in places where there is a considerably high density of Chinese ethnicity when compared to the native population, as in Durban and Gombak [17, 36].

No association was found between parental education and socio-economic status and hyperopia in children. As for ocular components, in the United States Lee observed a statistically significant association (p < 0.01) between years of education and larger AL in individuals aged 43-84 years, indicating that this aspect should be better studied in children [65].

Some authors point to geographical factors as potential determinants of ametropias, such as location and type of residence. They defend that greater levels of hyperopia may be found in people who live in rural areas and in houses, because they do more outdoor activities.

The controversy as to the impact of environmental factors on hyperopic spherical equivalent refraction in children still remains. Although theoretically near activities increase the demand of the accommodative process (hyperopic defocus), stimulating changes in the dimensions of ocular components (such as increases in AL) and thus decreasing the eye’s chance of remaining hyperopic [6], one cross-sectional study found very weak correlation between hours spent in near work activities and spherical equivalent [59]. Regarding outdoor activities, spending more time outdoors was associated with slightly more hyperopic refractions [59]. Theoretically, children who spend more hours per week doing outdoor activities do not require as much accommodation to practice them. Thus, the stimulation of ocular growth decreases owing to low accommodative demand [8]. The empirical evidence is insufficient to be able to understand the relationship between environmental factors and hyperopia.

The role of light intensity must also be considered. Since light is usually of greater intensity outdoors, eye exposure results in a more constricted pupil, increasing the depth of focus and leading to a less unfocused image [8]. In addition, dopamine released by light stimulus on the retina can contribute directly to inhibiting ocular growth [8, 66].

## Conclusion

The large variability of hyperopia prevalence raises questions about the ability of demographic, socio-economic and environmental factors to completely explain the hyperopia causal chain. Considering that more myopic populations or those with earlier onset of myopia may be populations with earlier or greater reductions in hyperopia, in view of the complementarity of these phenomena, the causes of the decrease in hyperopia prevalence may be common to those explaining the increase in myopia with age.

Future studies should refine the evaluation of these factors, particularly the role of outdoor activities and ethnicity, as well as exploring other potential risk factors such as heredity or diet. In order to improve the consistency of analysis, refractive error measurement needs to be standardized using the RESC Protocol and using cycloplegia to perform refractive examination. It is also important to have population-based or school-based representative samples, with low percentages of loss to follow-up and sufficiently large samples to be able to stratify by specific age. More studies on those younger than 9 years-old and with larger samples are necessary in order to obtain a more precise prevalence estimate.

AAO recommends undercorrection of hyperopia, however despite the fact that a large percentage of hyperopia appears to be benign at very early ages, a significant number may go on to develop sequelae. Furthermore, it is necessary to deepen the understanding about the interactions among hyperopic refractive error and accommodative and binocular functions as a way of identifying groups of hyperopic children at risk of developing visual, academic and even cognitive function sequelae [2].
